# The Physical and Psychological Benefits of Nordic Walking in Patients with Breast Cancer: A Systematic Review

**DOI:** 10.3390/medicina62050932

**Published:** 2026-05-11

**Authors:** Lucía Ortega-Pérez de Villar, Julio Fernández-Garrido, Omar Cauli

**Affiliations:** 1 Department of Nursing, Faculty of Nursing and Podiatry, University of Valencia, 46010 Valencia, Spain; lucia.ortega@uv.es (L.O.-P.d.V.); julio.fernandez@uv.es (J.F.-G.); 2 Cátedra Fundación Colisée de Envejecimiento Saludable, Activo y Participativo, University of Valencia, 46010 Valencia, Spain

**Keywords:** physical activity, quality of life, cardiovascular system, mental health, lymphedema

## Abstract

*Background and Objectives:* Breast cancer survivors often experience long-term physical and psychological impairments that negatively affect quality of life. Exercise-based rehabilitation strategies are increasingly recommended in this population. This study aimed to analyze the physical and psychological benefits of Nordic walking (NW) in breast cancer survivors. *Materials and Methods:* A systematic review (registered in PROSPERO ref: CRD42024613292) was conducted in accordance with PRISMA guidelines. SPORTDiscus, CINAHL, Cochrane, Embase, Medline Ovid and PubMed were searched from database inception to 30 December 2025, with no restrictions applied regarding the language of the included studies, using the terms “breast cancer”, “walking poles”, “pole walking” and “Nordic walking”. Studies involving adult women (≥18 years) with breast cancer were included. Data on study design, participant characteristics, intervention protocols and outcomes were extracted. Methodological quality was assessed using the Joanna Briggs Institute critical appraisal tools. *Results*: From 281 records identified, 17 studies were included that evaluated NW alone or in combination with other exercise. Improvements in cardiovascular capacity were reported in five studies (four out of five reported significant improvement after NW), while gains in shoulder range of motion were observed in three out of four studies assessing this outcome, mainly after standalone NW interventions. Positive changes in body posture were significantly reported after NW in all three studies investigating this parameter. Improvements in strength were reported in five studies, while gains in flexibility were observed in one study, within multimodal interventions combining NW with other exercise. Among seven studies evaluating lymphedema, sustained reductions were primarily reported after multiweek programs, especially when NW was combined with the ISA method. Several studies also reported improvements in pain, psychological alterations, and quality of life. *Conclusions*: NW appears to be an effective, holistic exercise modality for enhancing both physical and psychological aspects for breast cancer survivors.

## 1. Introduction

Breast cancer remains the most commonly diagnosed cancer in women worldwide. The latest GLOBOCAN 2022 estimates report about 2.3 million new female breast cancer cases and about 666,000 deaths globally [1,2]. Incidence is highest in more-developed regions, largely reflecting older populations, reproductive patterns, screening, and detection [3]. Mortality burden is disproportionately high in lower-resource settings because diagnosis often occurs later and access to treatment is less consistent [4]. In the United States, breast cancer incidence has been rising about 1% per year, especially for localized-stage and hormone-receptor-positive disease; the increase is also notable among women under 50 [5,6]. According to the Spanish Society of Medical Oncology (SEOM), breast cancer was the most diagnosed cancer in Spain in 2024. Although the net survival of women with breast cancer has increased in recent years [7], survivors must still combat the adverse effects of cancer treatment resulting from surgery, radiation, and/or chemotherapy that negatively affect their quality of life, especially on the physical and psychological levels. The most common side effects of these treatments include fatigue, insomnia, and strength impairment, amongst others [8,9,10].

The increase in adverse effects along with persistent symptoms over time leads to a higher level of sedentarism which in turn affects cardiorespiratory fitness and muscle strength, thereby limiting patient functionality and independence and impacting quality of life [11,12].

Cardiorespiratory problems are related to premature mortality, while a decline in strength is associated with functional deterioration, limiting independence and, consequently, reducing quality of life. In addition, psychological effects such as cognitive problems, depression, and anxiety are prevalent in this population. After chemotherapy, up to 60% of patients experience cognitive impairments and emotional distress, which also significantly impact their quality of life and contribute to feelings of depression and anxiety [10,13,14,15,16]. As a result, breast cancer patients may find themselves trapped in a vicious cycle, leading to decreased physical inactivity and further health complications [17,18,19,20].

The WHO guidelines on physical activity and sedentary behaviors for significant health benefits recommend that adults should accumulate a minimum of 150–300 min of moderate-intensity aerobic physical activity or a minimum of 75–150 min of vigorous-intensity aerobic physical activity throughout the week. Also, the equivalent of moderate- and vigorous-intensity activities can be combined to achieve significant health benefits [21]. Increased physical activity following a cancer diagnosis has been linked to a lower risk of all-cause mortality and cancer mortality.

Physical exercise has emerged as an important non-pharmacological strategy that can modulate several of these pathophysiological mechanisms [22,23,24,25,26]. Regular physical activity reduces circulating estrogen levels [22,27] and improves insulin sensitivity, thereby lowering insulin and insulin-like growth factor-1, both of which are implicated in tumor growth [22,26,27]. In addition, exercise exerts anti-inflammatory effects and enhances immune surveillance, contributing to a less favorable environment for cancer progression [25]. Evidence also suggests that exercise may improve tumor vascular function and oxygenation, potentially enhancing treatment efficacy [23,24,25].

Evidence reflects a nonlinear relationship between increasing levels of physical activity after diagnosis and breast cancer and all-cause mortality up to 10–15 MET-hours/week (which is equivalent to 150 min/week of moderate or vigorous physical activity), and there is no evidence of harmful effects at higher levels [28,29,30]. To accumulate this number of minutes of activity and thus avoid the effects of lack of physical exercise, the effectiveness of different exercise programs has been tested. These programs generally consist of aerobic or strength exercises, or a combination of both. Of note, taking part in an exercise program plays a key role in improving adherence to a regular exercise regimen, and so a wide range of methods to increase participation and compliance have been developed. Thus, to promote participation, researchers have sought activities that are socially and physically attractive [31,32,33].

Nordic walking (NW), which combines elements of classic walking, skiing, and trekking, is one of these activities. It has proven to be a simple form of physical activity that can be performed by almost anyone, anywhere. NW has similar characteristics to power walking (brisk walking) except for the additional use of two poles specifically designed to activate the upper body musculature, providing added value from a physiological and biomechanical point of view, with health benefits substantially superior to power walking [34]. Furthermore, NW provides a full-body workout that engages both the upper and lower body, activating approximately 70–90% of the body musculature and resulting in an 8% increased energy expenditure compared to regular walking, along with a reduction in joint load by approximately 30%. It has also been shown to increase gait speed and improve cardiovascular function [35,36,37].

The characteristics of NW particularly benefit breast cancer survivors, including improved aerobic capacity, strength, balance, and well-being, both in healthy individuals and those with various conditions or diseases [34,37]. It positively impacts the cardiorespiratory system, improves lipid profiles, promotes weight loss, mitigates sarcopenia, and helps alleviate pain [37]. The combination of open and closed kinetic chain movements in the upper limbs during NW creates muscle contractions that facilitate a pumping effect, enhancing circulation of both lymph and blood through the arms [38]. Furthermore, physiological studies found significant differences during NW compared to normal walking, such as increased heart rate, oxygen consumption (VO_2_), caloric expenditure, and lactic acid concentration [34].

From a biomechanical point of view, NW improves stability because it ensures an enlarged base of support, increased stride length, contact time, and execution speed; it decreases compression forces on the knees and reduces vertical ground reaction forces and pressure on the central metatarsal [35]. Furthermore, increasing the intensity of arm movement with the poles intensifies upper body muscle strength and reduces shoulder and neck pain by up to 40% compared to people with chronic neck pain who do not do MN exercise [34], all without significantly increasing the subjective perception of exertion [37]. These characteristics make NW a simple, safe, effective, enjoyable, and low-cost physical activity and so it is likely particularly beneficial to breast cancer patients [39,40]. A previous review by Sánchez-Lastra et al. [41] examined the effects of Nordic walking (NW) in breast cancer survivors, including nine studies focused on physical symptoms such as lymphedema, physical fitness, disability and morbid perceptions. Given the growing body of evidence on this topic, we conducted an updated systematic review providing a more comprehensive synthesis. This review expands upon previous work by analyzing the effects of NW on physical function components, and specifically, cardiovascular capacity, joint mobility, posture, flexibility, and strength along with its impact on lymphedema, pain, quality of life, and psychological alterations.

## 2. Materials and Methods

This systematic review was conducted according to the Preferred Reporting Items for Systematic Reviews and Meta-Analyses (PRISMAs) guideline. The selected search strategy and methods of analysis were registered at the PROSPERO database (ref: CRD42024613292).

### 2.1. Literature Search

A literature search using multiple electronic bibliographic databases was conducted between September 2025 and 30 December 2025. SPORTDiscus, CINAHL, Cochrane, Embase, Medline Ovid, and PubMed were searched from database inception to 30 December 2025, with no restrictions applied regarding the language of the included studies. The last search was conducted on 30 December 2025. The primary search terms used were “breast cancer” AND (“walking poles” OR “pole walking” OR “Nordic walking”). No restrictions were applied regarding publication date or language of the included studies. The complete search strategies for each database, including Boolean operators, are provided in the Supplementary File.

### 2.2. Inclusion/Exclusion Criteria

The selection criteria for the systematic review were defined according to the PICOS framework (population, intervention, comparison, outcomes, and study design criteria) [42]. Studies were included if they presented data from baseline and at least one post-intervention, and/or short, medium and/or long-term follow-up assessment.

#### 2.2.1. Population

Female adult patients (≥18 years) diagnosed with breast cancer, regardless of the cancer type, stage, or oncological treatment.

#### 2.2.2. Intervention and Comparison

Studies evaluating NW as standalone intervention or NW combined with other exercise modalities (e.g., aerobic training, resistance training), compared with usual care, other exercise interventions, or pre-post-intervention comparisons.

#### 2.2.3. Outcomes

Studies reporting at least one physical or psychological outcome related to NW, including cardiovascular capacity, range of motion, body posture, flexibility, muscular strength, lymphoedema, pain, quality of life, psychological well-being or cognitive outcomes.

#### 2.2.4. Studies’ Design

Randomized controlled trials (RCTs), including pilot studies, quasi-experimental studies, and uncontrolled studies, were included. The inclusion of different study designs was justified by the limited availability of RCTs in this field. No restrictions were applied regarding publication date or language.

#### 2.2.5. Exclusion Criteria

Studies were excluded if they: (i) included male participants or pediatric/adolescent populations; (ii) did not involve NW as part of the intervention; (iii) were qualitative studies, conference abstracts, protocols, editorials, or narrative reviews; or (iv) did not report data regarding physical and/or psychological outcomes.

### 2.3. Analysis and Synthesis

The citation list of all articles was imported to an online citation manager (Zotero5.0.85 (http://www.zotero.org)) which was used to manage the screening process and remove duplicate citations. In the first phase, we assessed the relevance of the studies in relation to the study questions and objectives using information from the study title, abstract, and keywords, assessing the full text where there was no consensus or insufficient information. In the second phase, we assessed the full text of each study for compliance with the inclusion criteria. The article selection process was conducted and evaluated by 2 independent researchers (L.O.P.d.V. and J.F.G.) and differences were resolved by consensus, moderated by a third researcher (O.C.) [43]. No automation tools were used at any stage of the selection or data extraction processes. Two independent researchers (L.O.P.d.V. and J.F.G.) extracted study characteristics and outcome data, ensuring that the most relevant information was obtained from each study [44], as well as the country in which the study was conducted, number of participants, participant characteristics, NW programs, dropouts, and outcomes. Results related to the effect of exercise training were extracted from the post-intervention and/or follow-up assessments. A quantitative meta-analysis was not considered appropriate due to substantial clinical and methodological heterogeneity across the included studies. Specifically, marked variability was observed in intervention characteristics (standalone NW versus NW combined with other exercise modalities), intervention duration and frequency, comparator conditions, and outcome measures. In addition, outcomes were assessed using different measurement tools and reported using heterogeneous metrics, which precluded meaningful statistical pooling of effect sizes. Furthermore, the included studies encompassed different methodological designs, including RCTs, quasi-experimental studies, and uncontrolled intervention studies, contributing to additional heterogeneity in study quality and risk of bias. For these reasons, a structured narrative synthesis was conducted in accordance with current methodological recommendations for systematic reviews when meta-analysis is not feasible [45]. This approach typically organizes results thematically according to key characteristics, such as intervention type, population group, or outcome domain.

### 2.4. Quality Appraisal

The quality assessment of the studies to be included in this review followed the critical appraisal tools of the Joanna Briggs Institute (JBI), which help assess the reliability, relevance, and results of published articles, and the checklist was completed according to the type of study. JBI offers a suite of critical appraisal instruments that are freely available to systematic reviewers and researchers investigating the methodological limitations of primary research studies. The JBI instruments are designed to be study-specific and are presented as questions in a checklist. In our case we used the checklist for quasi-experimental [46] and RCT studies [47].

### 2.5. Overall Strength of the Evidence

We evaluated the certainty of evidence by classifying the results according to the levels defined in the Grading of Recommendations, Assessment, Development and Evaluation (GRADEs) framework [48]. Two researchers (L.O.P.d.V. and J.F.G.) carried out this evaluation, taking into account the following 5 key domains adapted for network meta-analyses: risk of bias, inconsistency, indirectness, imprecision and publication bias. Due to the substantial heterogeneity in the exercise modalities across the included studies a meta-analysis was not feasible. The level of evidence was categorized as high, moderate, low, or very low quality of evidence.

## 3. Results

**Figure 1:**
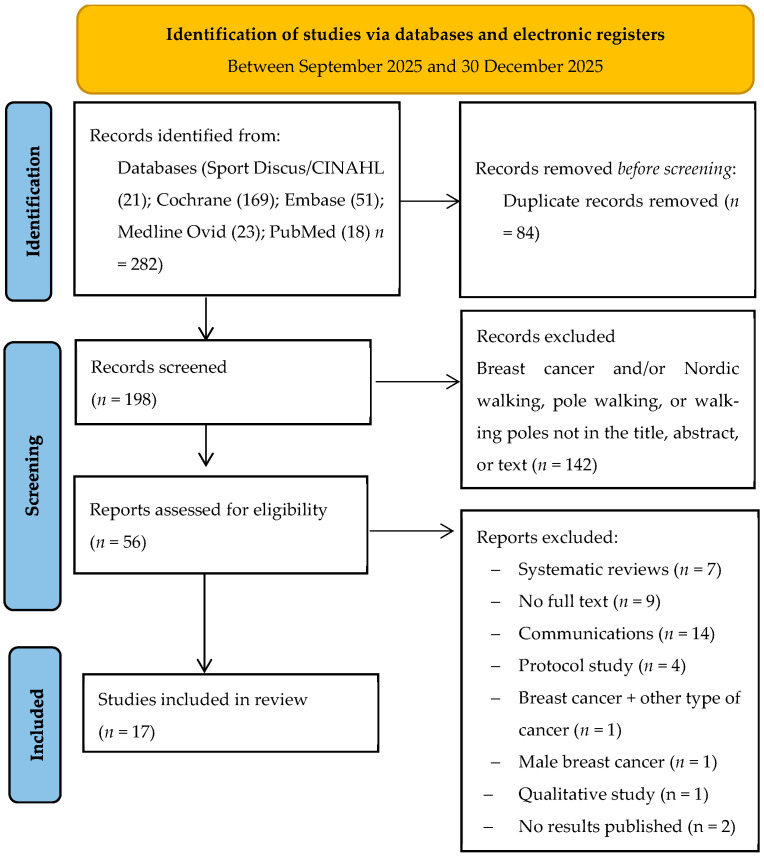
PRISMAs (Preferred Reporting Items for Systematic Reviews and Meta-analyses) flow diagram.

Our electronic search strategy retrieved 282 studies, of which only 17 met the inclusion criteria and were included in the final analysis. The PRISMA flow diagram illustrating the number of studies excluded at each stage of the systematic review is shown in Figure 1.

The JBI levels of evidence for the studies were as follows: eight studies provided level 1 evidence (studies with experimental designs) (Table 1) and six provided level 2 evidence (studies with quasi-experimental designs) (Table 2) for 13 and nine items, respectively. The evaluation criteria were based on the items marked as “yes” (scored as ‘1’) and “unclear” or “no” (scored as ‘0’). The higher the score, the better the quality of the study.

### 3.1. Study Characteristics

The 17 selected studies were conducted between 2005 and 2025 and enrolled a total of 1080 adult women diagnosed with breast cancer, with a mean age of 56.67 years. Sample sizes across the studies ranged from 181 [52] to 16 patients [50,55]. Meta-analysis was not possible because of the incompatibility of the outcome measures amongst the studies as detailed below.

### 3.2. Intervention Classification

Intervention duration ranged from a single NW session [63] to structure training programs lasting up to 6 months [52,53], with prescribed frequency varying from once per week [54,62] to five sessions per week [60].

Interventions were categorized into the following four subgroups based on the structure of the exercise programs: (1) NW as a standalone intervention [49,50,51,56,58,59,60,62,63,65], (2) NW combined with resistance training and/or concurrent conditioning exercises [54,55,64], (3) NW with structured aerobic and multicomponent supervised training [52,53,57] and (4) NW combined with ISA method or myofascial exercise method [50,51,61].

Table 3 summarizes the characteristics of each intervention used in the selected studies. The ISA technique is a set of dynamic exercises created especially for breast cancer survivors that are therapeutic for arthralgia, lymphoedema, and NW. This calls for the use of ISA balls, which are foam balls with a diameter of 6 or 7 cm and varying densities that can be used on their own or in conjunction with NW poles. The exercises in the series are designed to counteract or prevent upper limb lymphoedema, ease muscle tension, and gently warm up the joints. The training program began with hand and wrist joint workouts that were done solely with ISA balls. Following these, multi-joint exercises (such as those for the wrists, elbows, and shoulders) were performed with the NW poles and ISA balls.

### 3.3. Organization of the Main Outcomes

The effects of NW training were summarized based on the reported benefits in physical function outcomes, including cardiovascular capacity (five studies), range of motion (four studies), body posture (three studies), flexibility, and strength (five studies), on lymphoedema (seven studies) and, in reducing pain, improving quality of life, in psychological aspects and cognitive functioning (six studies).

#### 3.3.1. Shoulder Motion and Morbidity

Four studies [55,57,62,64] analyzed the range of motion in the shoulder joint following NW-based interventions with different training structures. Overall, three of the four studies reported significant improvements in shoulder mobility, while one study reported no significant changes. Specifically, Sprod et al. [55] did not observe significant changes after an 8-week intervention combining NW with concurrent aerobic and resistance training. In contrast, studies evaluating standalone NW interventions consistently reported positive effects. Fischer et al. [62] reported significant improvements in both active (*p* < 0.01) and passive (*p* < 0.05) movements of the affected shoulder after 10 weeks of standalone NW. These findings are also supported by Vuckovic et al. [64] who observed significant increases in shoulder anteflexion, retroflexion and abduction after 10 weeks of NW program. Additionally, Casanovas-Álvarez et al. [57] demonstrated that a pre-rehabilitation program including NW was effective in maintaining arm function and preventing the decline in mobility typically observed during neoadjuvant treatment. This study also reported significant improvements in shoulder morbidity in terms of both pain and disability, with greater reduction from post-intervention to follow-up (T2–T3) than from pre- to post-intervention (T1–T2). Shoulder symptoms and limitations in activities of daily living (ADL) significantly improved across both time intervals (see Supplementary Tables S1–S4).

#### 3.3.2. Body Posture

Three studies examined trunk muscle endurance and postural alignment following standalone NW interventions [56,58,59]. Overall, all three studies reported significant improvements in at least one parameter related to trunk muscle function or postural alignment. Hanuszkiewicz et al. measured trunk functional and postural changes in women after breast cancer [56] or during treatment [58,59]. After 8 weeks of NW, a significant increase in both total work and average power of the trunk flexor and extensor muscle was observed in middle-aged women (45–59 years old), whereas only flexor muscle endurance improved significantly in older women (≥60 years old). No significant changes in trunk function or postural parameters were observed in the group performing general gymnastic exercises. In studies comparing NW with water exercise and general fitness [58,59] significant intra- and intergroup improvements in trunk function [58] and postural changes [59] were found exclusively in the NW and water exercise group (see Supplementary Tables S1–S4).

#### 3.3.3. Flexibility, Strength, and Cardiovascular Fitness

Only one study was found related to flexibility in female breast cancer survivors: Morano et al. [61] assessed shoulder, lower back, and hamstring flexibility following 12 weeks of NW combined with the ISA method and reported significant improvements in both upper and lower back flexibility (see Supplementary Tables S1–S4). As this was a combined intervention, flexibility gains cannot be attributed to NW alone.

Muscle strength was evaluated in five studies using different training structures and assessment tools. [54,55,57,61,64] Four of the five studies reported significant changes. Morano et al. [61] and Vuckovic et al. [64] reported significant improvements in forearm strength and the non-dominant hand; conversely, Casanovas-Álvarez et al. [57] did not observe significant changes following their pre-rehabilitation program. Furthermore, Vuckovic et al. [64] provided evidence on lower-body functional strength through the STS-30, reporting a significant increase in the number of repetitions (*p* < 0.001), which reflects improvement in overall functional power.

In terms of muscular endurance, Morano et al. [61] observed significant gains in trunk extensor endurance (single leg back bridge test). Similarly, Sprod et al. [55] and Malicka et al. [54] assessed upper limb muscular endurance following NW combined with concurrent aerobic and resistance conditioning. Sprod et al. [55] reported significant increase in bench press and latissimus dorsi pull-down repetitions after 8 weeks, whereas Malicka et al. [54] found significant gains in a pushing-and-pulling motion measured by isokinetic dynamometer. Overall, while consistent strength improvements were observed across studies, all positive findings derived from multimodal exercise protocols incorporating NW, precluding firm conclusions regarding the isolated effects of NW on muscular strength.

Four studies (reported across five studies) [52,53,57,60,64] evaluated cardiovascular capacity (see Supplementary Tables S1–S4). Three of the four studies reported significant improvements in cardiovascular outcomes. Using a submaximal bicycle ergometer test, Jönsson and Johansen [60] reported a significant decrease in heart rate in the intervention group after 8 weeks of standalone NW (mean difference = −5 bpm; *p* = 0.004). In turn, Koevoets et al. [52,53] observed a significant increase in a maximal aerobic capacity from baseline to follow-up in the intervention group compared with the control group, as assessed by a maximal-cycle cardiopulmonary exercise test using a ramp protocol with continuous breathing gas analysis and electrocardiogram monitoring. Consistent with these findings, both Casanovas-Álvarez et al. [57] and Vuckovic et al. [64], reported a significant improvement in functional capacity measured via the 6 MWT, further highlighting NW as a safe and effective modality for enhancing aerobic fitness in breast cancer survivors.

#### 3.3.4. Lymphoedema

Lymphoedema was evaluated in seven studies [50,51,54,57,60,63,64]. Overall, sustained reductions in lymphoedema-related outcomes were reported in three studies, whereas four studies reported null or transient effects. Regarding acute effects, one study assessing a single session reported a transient reduction in both absolute and relative volume immediately after NW alongside a significant increase in the volume of the healthy arm; however, all lymphedema-related changes did not remain 24 h later [63]. In contrast, studies implementing multiweek NW programs showed more consistent medium-term effects. After 8 weeks of standalone NW, Jönson and Johanson [60] reported significant reductions in total arm volume absolute and relative lymphedema volume. However, several studies [54,57,64] did not observe significant volume reductions following a combined NW and concurrent conditioning program. Furthermore, in studies incorporating NW with the ISA method, significant reductions in upper-limb circumferences, extracellular body water, and the extracellular-to-total body water ratio were consistently observed after 10 weeks of training [50,51]. Overall, sustained reductions in lymphedema-related parameters were primarily associated with repeated multiweek interventions, particularly when NW was combined with the ISA method (Table 4).

#### 3.3.5. Psychological Aspects, Cognitive Functioning, Pain, and Quality of Life

Psychological outcomes, cognitive functioning, pain, and quality of life were assessed in six studies with different intervention structures [49,52,53,57,64,65]. Overall, psychological well-being, pain, and quality of life outcomes showed predominantly positive effects, whereas cognitive outcomes showed largely null or heterogeneous findings.

Psychological well-being was assessed in two studies and showed significant improvements in response to exercise programs. Koevoets et al. [52] reported a significant reduction in depressive symptoms (PHQ-9) after a multicomponent exercise program including NW, although no significant between-group differences were found for anxiety. Similarly, Fields et al. [49] observed improvements in psychological distress in both the NW and usual care groups, with a trend toward greater enhancement in the NW arm.

Cognitive functioning was evaluated in two studies. Koevoets et al. [52] found no significant changes in global cognitive performance after 6 months of training. Neuroimaging data from the same cohort showed no significant differences in total hippocampal volume or gray matter volume between groups [53]. However, regional analysis revealed that volumetric reductions in the right hippocampus, dentate gyrus, and subiculum were paradoxically associated with improvements in cognitive functioning, suggesting a complex compensatory mechanism or structural remodeling [53].

Pain outcomes were evaluated in three studies [49,57,64], all of which reported significant improvements following NW interventions. After 6 weeks, Fields et al. [49] reported a clinically significant reduction (30%) in worst pain intensity among women with AIAA after 6 weeks of standalone NW, which was maintained at the 12-week follow-up. This is further supported by more recent evidence from Casanovas-Álvarez et al. [57], who reported a significant decrease in pain intensity (*p* = 0.04) following an 8-week supervised NW-based prehabilitation program in patients receiving neoadjuvant chemotherapy. Similarly, Vuckovic et al. [64] found that a structured NW intervention led to a significant reduction in both pain scores and the sensation of heaviness in the affected limb. Collectively, these findings suggest that NW is an effective and safe non-pharmacological strategy for mitigating treatment-related pain and physical discomfort in breast cancer survivors.

Quality of life was evaluated in five studies [49,52,57,64,65] and improved significantly across the interventions, reflecting enhancements in functional capacity and the mitigation of cancer-related symptoms. Fields et al. [49] observed that NW practice led to improvements across all SF-36 subscales, notably preventing the deterioration in general health perception and physical functioning seen in control groups. Koevoets et al. [52] and Casanovas-Álvarez et al. [57] also reported significant gains in global health status, physical functioning, and role functioning, alongside a marked reduction in fatigue.

Sartor et al. [65] provided critical evidence on the physical recovery of survivors, demonstrating that an aerobic program incorporating NW successfully reduced sedentary time and significantly improved sleep efficiency. Furthermore, Vuckovic et al. [64] linked the increased functional capacity achieved through NW with greater autonomy in daily activities, further consolidating the positive impact of this modality on the overall perceived well-being of breast cancer survivors.

### 3.4. Quality of Evidence

The grading of the quality of evidence using GRADE took into account imprecision and indirectness; therefore, the certainty of evidence ranged from low to very low and the results should be interpreted with caution (see Table 5).

## 4. Discussion

In recent years, NW has been proposed as an effective and accessible rehabilitation option for breast cancer survivors due to its versatility and because it can be performed anywhere, either individually or in groups. This type of exercise provides positive effects both physically and psychologically in this group of patients. Therefore, this systematic review synthesized the available evidence on the effects of NW in female breast cancer survivors, focusing on physical, cardiovascular, lymphatic, and psychological outcomes.

One of the most characteristic and debilitating side effects of breast cancer treatment is its impact on the shoulder on the affected side, which often leads to reduced mobility and increased morbidity, especially after surgery and radiotherapy [55]. The improvement on shoulder mobility seems to be due a combination of biomechanical, neuromuscular, and circulatory mechanisms [54,55]. The use of poles during NW promotes active engagement of the upper limbs, encouraging repetitive shoulder flexion, extension, and coordinated scapulothoracic movement, which can counteract post-surgical stiffness and adhesions commonly observed after mastectomy or radiotherapy [55]. This dynamic loading enhances muscle strength in the deltoids, rotator cuff, and scapular stabilizers while simultaneously facilitating proprioceptive feedback and motor control re-education. [66]. Additionally, the rhythmic, low-impact nature of NW improves lymphatic drainage and reduces the risk of lymphedema by stimulating muscle pump activity, thereby decreasing swelling and allowing greater joint range of motion [67]. Improved posture and trunk rotation associated with NW further contribute to restoring functional shoulder kinematics and reducing compensatory movement patterns. Collectively, these adaptations support both structural and functional recovery of the shoulder complex in breast cancer [68]. Improvements in shoulder mobility and shoulder-related disability were observed only after longer standalone NW interventions (10 weeks) [62], whereas shorter interventions and combined protocols did not yield consistent benefits [55]. This suggests a potential dose–response relationship, although conclusions remain tentative due to the small number of trials and variation in intervention structure.

Age-related differences were also evident in trunk muscle and postural adaptations, with middle-aged women showing greater gains than older participants, highlighting the need for individualized training prescriptions based on functional capacity and age [56,58,59]. Breast cancer treatments, especially surgery, radiotherapy, and chemotherapy, negatively affect muscle function, causing weakness, atrophy, and stiffness [54]. Consistent improvements in muscular strength and flexibility were observed exclusively in studies employing multimodal exercise protocols in which NW was combined with resistance training or the ISA method [54,55,61]. This may be because of the muscle activation required by this type of exercise but nevertheless, more work will be needed to confirm these results. Unlike regular walking, NW incorporates upper-limb propulsion via poles, leading to greater activation of the shoulder girdle, trunk stabilizers, and arm musculature, which is particularly beneficial for patients recovering from surgery or affected by treatment-related deconditioning [55]. This full-body involvement increases muscle strength by promoting resistance-like loading in both upper and lower extremities while maintaining low joint impact [34,69]. Additionally, the coordinated, rhythmic movement patterns improve range of motion and joint mobility, especially in the shoulders, thereby enhancing flexibility and reducing stiffness often associated with post-surgical scarring or radiation therapy [61]. Furthermore, regular practice induces anti-inflammatory effects and improves circulation, which may facilitate tissue elasticity and recovery [68,70].

Cardiorespiratory function, which is associated with premature mortality, is another of the main issues affecting breast cancer survivors [31]. Standalone NW interventions improved submaximal cardiovascular efficiency, as reflected by reduced heart rate during standardized exercise testing, whereas multicomponent exercise programs combined with NW interventions contribute to significant improvements in maximal aerobic capacity (VO_2_ peak). These findings indicate that NW may contribute to cardiovascular conditioning, although magnitude of benefit appears dependent on training intensity, supervision, and whether NW is delivered as a standalone or multicomponent intervention [52,53,60].

Similarly, NW has also shown positive effects in reducing lymphedema, a very common complication following breast cancer surgery. Acute single-session NW produced only transient reductions in arm volume [63], whereas repeated multiweek interventions yielded more consistent medium-term reductions in arm circumference and extracellular fluid, particularly when NW was combined with ISA method or myofascial techniques [50,51,54,60]. This may be because of the dynamic movement resulting from use of the poles, which facilitates drainage and promotes circulation during walking. Furthermore, lymphatic drainage in the arms may occur as the result of hand contractions, aiding in the removal of excess fluid in this area [71].

Regarding the psychological effects of breast cancer, cognitive decline is a common concern among patients who have undergone chemotherapy, which can lead to issues with memory, concentration, and mental processing [72,73,74]. Although some studies suggest that exercise programs, including NW, may have beneficial effects on cognitive function, we found no statistically significant changes had been reported in this sense [52,53]. Thus, psychological outcomes varied depending on the type and duration of intervention. Similarly, depression, pain, and reduced quality of life are also common among breast cancer survivors [75,76,77]. In this context, it has been demonstrated that NW can improve not only physical function but also the perception of well-being in this group, thereby reducing levels of anxiety, stress, and depression [34]. Moreover, participation in NW is commonly performed in group settings, fostering social interaction, companionship, and emotional support, which can enhance adherence and psychosocial well-being among participants [78,79]. In the analyzed studies, NW seemed to improve the psychological state and quality of life of these patients, which is crucial for their recovery [49,52], although comparison with proper control group(s) needs additional studies.

Several limitations of this review must be acknowledged. Although NW entails favorable changes in the physical and psychological symptomatology that breast cancer survivors show, according to the observed results in the examined studies, these findings should be interpreted cautiously for several reasons. First, there were not many research studies available, and the quality of their methodology was not high based on GRADE critical appraisal [48]. Second, the studies used small sample numbers and did not perform an estimate analysis on the sample sizes, which restricts the statistical significance and raises the possibility of error type II. Lastly, just few studies carried out a precise follow-up phase, which restricts our understanding of how the variables behave over time. No meta-analysis was carried out because of the significant variation in intervention parameters, outcome measures, and data reporting techniques. Selective publishing and reporting in the literature, which is another significant source of bias, might be another restriction. Lastly, the lack of a review of the gray literature may have limited coverage of all possible research on this subject. Most studies were characterized by small sample sizes, lack of blinding, incomplete reporting of intervention dosage, and heterogeneity in both interventions and outcomes. In addition, only a minority of studies employed randomized controlled designs, and long-term follow-up data were scarce. These methodological weaknesses substantially reduce the certainty of the evidence. Furthermore, outcome measures and assessment tools differ widely across studies, limiting direct comparisons and precluding quantitative meta-analysis. Clear differentiation between standalone NW and multimodal exercise programs is essential to allow attribution of effects. Studies should also report clinically meaningful thresholds, include long-term follow-up, and explore dose–response relationships. Further research is particularly warranted to clarify the effects of NW on cognitive outcomes, lymphedema progression, and age-specific musculoskeletal adaptations.

Future demands for NW in breast cancer survivors would center on its integration into standard oncology care as a prescribed, evidence-based intervention, rather than an optional activity. This includes the development of structured referral pathways from oncologists to exercise professionals and the incorporation of Nordic walking into survivorship care plans. There will also be a growing need for personalized exercise prescriptions, adapting intensity, frequency, and technique to individual patient characteristics such as treatment stage, comorbidities, and functional limitations. In parallel, the expansion of digital health solutions, including wearable monitoring and tele-rehabilitation platforms, will be essential to improve accessibility and long-term adherence. Future demands will further require large-scale, high-quality clinical trials to establish optimal training protocols and clarify long-term outcomes such as recurrence and survival. Finally, the implementation of community-based and group programs will be critical to support behavioral change, enhance psychosocial well-being, and ensure sustainability of physical activity among breast cancer survivors.

NW has several practical clinical applications in breast cancer survivorship care, functioning as a safe and effective form of structured exercise that can be integrated into rehabilitation programs. It can be prescribed in the early post-treatment phase to improve cardiorespiratory fitness, muscle strength, and functional capacity, while also helping to reduce cancer-related fatigue. The use of poles engages the upper body, which may support shoulder mobility and contribute to lymphatic flow, making it potentially beneficial for women at risk of or living with lymphedema when appropriately supervised. In addition, Nordic walking is particularly useful for improving cardiometabolic health. The psychological benefits have started to be described and its effects on psychological alterations warrant future clinical trials.

## 5. Conclusions

Studies about rehabilitation in breast cancer survivors suggest that NW improves cardiorespiratory fitness and muscle strength, reduces cancer-related fatigue, enhances quality of life and psychological well-being and improves lymphedema when properly supervised. Its inclusion in survivorship care aligns with broader exercise-oncology recommendations emphasizing aerobic plus resistance training. Overall, NW represents a low-cost, accessible, and scalable intervention that aligns well with current exercise-oncology recommendations for comprehensive survivorship care. Nevertheless, further research with control group is still needed to improve intervention protocols and to assess the long-term effects of NW, and particularly mental health effects should be further investigated.

## Figures and Tables

**Table 1:** Critical appraisal of the experimental studies included in this review (JBI Critical Appraisal Checklist for Randomized Controlled Trials).

Reference	Q1	Q2	Q3	Q4	Q5	Q6	Q7	Q8	Q9	Q10	Q11	Q12	Q13	%Y
Fields et al. [49]	Y	Y	Y	N	N	N	Y	Y	N	Y	Y	Y	Y	69.23
Di Blasio et al. [50]	UC	N	Y	N	N	N	Y	Y	N	Y	Y	Y	Y	53.85
Di Blasio et al. [51]	UC	N	Y	N	N	N	Y	Y	N	Y	Y	Y	Y	53.85
Koevoets et al. [52]	Y	Y	Y	N	N	N	Y	Y	Y	Y	Y	Y	Y	76.92
Koevoets et al. [53]	Y	Y	Y	N	N	N	Y	Y	Y	Y	Y	Y	Y	76.92
Malicka et al. [54]	Y	N	Y	N	N	N	Y	Y	N	Y	Y	Y	Y	61.54
Sprod et al. [55]	Y	N	Y	N	N	N	Y	Y	N	Y	Y	Y	Y	61.54
Hanuszkiewicz et al. [56]	Y	N	Y	N	N	N	Y	Y	N	Y	Y	Y	Y	61.54
Casanovas-Álvarez et al. [57]	Y	Y	Y	N	N	Y	Y	Y	Y	Y	Y	Y	Y	84.61%

Abbreviations: Y, yes; N, no; UC, unclear. JBI Critical Appraisal Checklist for Randomized Controlled Trials: Q1 = “Was true randomization used for assignment of participants to treatment groups?”; Q2 = “Was allocation to treatment groups concealed?”; Q3 = “Were treatment groups similar at the baseline?”; Q4 = “Were participants blind to treatment assignment?”; Q5 = “Were those delivering treatment blind to treatment assignment?”; Q6 = “Were outcomes assessors blind to treatment assignment?”; Q7 = “Were treatment groups treated identically other than the intervention of interest?”; Q8 = “Was follow up complete and if not, were differences between groups in terms of their follow up adequately described and analyzed?”; Q9 = “Were participants analyzed in the groups to which they were randomized?”; Q10 = “Were outcomes measured in the same way for treatment groups?”; Q11 = “Were outcomes measured in a reliable way?”; Q12 = “Was appropriate statistical analysis used?”; Q13 = “Was the trial design appropriate, and any deviations from the standard RCT design (individual randomization, parallel groups) accounted for in the conduct and analysis of the trial?”.

**Table 2:** Critical appraisal of the non-experimental studies included in this review (JBI Critical Appraisal Checklist for Quasi-Experimental Studies, Non-Randomized Experimental Studies).

**Reference**	**Q1**	**Q2**	**Q3**	**Q4**	**Q5**	**Q6**	**Q7**	**Q8**	**Q9**	**%Y**
Hanuszkiewicz et al. [58]	Y	Y	N	N	Y	Y	Y	Y	Y	77.78
Hanuszkiewicz et al. [59]	Y	Y	N	N	Y	Y	Y	Y	Y	77.78
Jönsson and Johansson [60]	Y	NA	NA	N	Y	Y	NA	Y	Y	55.55
Morano et al. [61]	Y	N	N	N	Y	Y	Y	Y	Y	66.66
Fischer et al. [62]	Y	NA	NA	N	Y	Y	NA	Y	Y	55.55
Jönsson and Johansson [63]	Y	NA	NA	N	Y	Y	NA	Y	Y	55.55
Vuckovic et al. [64]	Y	NA	NA	N	Y	Y	Y	Y	Y	66.66
Sartor et al. [65]	Y	N	N	NA	Y	NA	Y	Y	Y	55.55

Abbreviations: Y, yes; N, no; NA, not applicable JBI Critical Appraisal Checklist for Quasi-Experimental Studies: Q1 = “Is it clear in the study what is the “cause” and what is the “effect” (i.e., there is no confusion about which variable comes first)?”; Q2 = “Were the participants included in any comparisons similar?”; Q3 = “Were the participants included in any comparisons receiving similar treatment/care, other than the exposure or intervention of interest?”; Q4 = “Was there a control group?”; Q5 = “Were there multiple measurements of the outcome both pre and post the intervention/exposure?”; Q6 = “Was follow up complete and if not, were differences between groups in terms of their follow up adequately described and analyzed?”; Q7 = “Were the outcomes of participants included in any comparisons measured in the same way?”; Q8 = “Were outcomes measured in a reliable way?”; Q9 = “Was appropriate statistical analysis used?”.

**Table 3:** Main characteristics of the interventions used in the selected studies.

Reference	Sample Characteristics (Number of Patients (Intervention/Control), Mean Age (Standard Deviation)	Program Duration	Frequency	Intensity	Nw Standalone/ Combine	Variable	Main Outcomes
Fields et al. [49]	*n* = 512 (20/20) Mean age = 63 (8)	6 weeks supervised NW + 6 weeks independent NW	1 h/w to 4 × 30 min sessions/w	Borg scale 11 to 14	Standalone NW	Psychological aspects Pain	CES-D BPI PSEQ
Di Blasio et al. [50,51]	*n* = 16 (8/8) Mean age = 49.09 (2.24)	10 lessons	3 t/week	Borg scale 10–14	Standalone NW NW + ISA method	Lymphoedema	Forearm circumferences → anthropometric tape
Di Blasio et al. [50,51]	*n* = 20 Mean age= 50.60 (3.60)	10 lessons	3 t/week	Borg scale 10–14	Standalone NW NW + ISA method	Lymphoedema	Forearm circumferences → anthropometric tape
Koevoets et al. [52]	*n* = 3258 (91/90) Mean age = 52.1 (8.6)/52.5 (8.7)	6 months	4 h/week	Individualized	NW+ multicomponent	Psychological aspects Cardiovascular	PHQ-9 HADS VO_2_ peak EORT QLQ-C30
Koevoets et al. [53]	*n* = 3258 (70/72) Mean age = 52.5 (9.9)/53.2 (8.6)	6 months	2 t/week	55–65% of the HR reserve	NW+ multicomponent	Cardiovascular Cognitive function QoL	Cardiopulmonary exercise test Hippocampal volume Global gray matter changes Memory functioning → Wordlist Learning trial of the ACS MRI Cognitive functioning → Hopkins Verbal Learning Test Revised (HVLT-R total recall)
Malicka et al. [54]	*n* = 38 (23/15) Mean age = 62.8 (6.1)	8 weeks	2 t/week	85% of max HR	NW + concurrent conditioning	Strength lymphoedema	Biodex multi joint Volume of lymphoedema: 5 levels from the line set by the ulnar styloid process to the radii, continuing every 10 cm up to the shoulder
Sprod et al. [55]	*n* = 16 (6/6) Mean age = 50.33 (2.74)/59.17 (4.62)	8 weeks	2 t/week	40–50% of heart rate reserve	NW + concurrent aerobic and resistance training	Shoulder motion and morbidity Strength	Goniometer Bench press Shoulder press Latissimus pull down
Hanuszkiewicz et al. [56]	*n* = 58 (19/20) Mean age = 58.8 (7.30)	8 weeks	2 t/week	65–70% maximum heart rate	Standalone NW	Body posture	Dynamometer isokinetic Biodex multi joint CQ Elektronik System
Hanuszkiewicz et al. [58]	*n* = 60 (NW = 20/GE = 20/WE = 20) Mean age: NW = 57.3 (8.05) GE = 59.4 (7.47) WE = 63.0 (7.58)	8 weeks	2 t/week	70–75% max HR	Standalone NW	Body posture	Dynamometer isokinetic Biodex multi joint
Hanuszkiewicz et al. [59]	*n* = 60 (NW = 20/GE = 20/WE = 20) Mean age: NW = 57.3 (8.05) GE = 59.4 (7.47) WE = 63.0 (7.58)	8 weeks	2 t/week	70–75% max HR	Standalone NW	Body posture	Photogrammetric body posture test
Jönsson and Johansson [60]	*n* = 35 (23/no control group) Mean age = 60.4 (8.3	8 weeks	3/5 t/week 30–60 min	70–80% of HR max	Standalone NW	Cardiovascular Lymphoedema	Bicycle ergometer test (heart rate) TAV (mL) LAV (mL) LRV (%) VAS (mm)
Morano et al. [61]	*n* = 160 (NW = 49/ME = 70) Mean age = 52.85 (7.26)	12 weeks	3 t/week	Borg 12–14	NW + ISA method	Flexibility Strength	Back scratch test Sit and reach test Single leg back bridge test Handgrip
Fischer et al. [62]	*n* = 77 (28/no control group) Mean age = 53.8 (10.0)	10 weeks	1 t/week	NR	Standalone NW	Shoulder motion and morbidity	Goniometer SPADI BIPQ
Jönsson and Johansson [63]	*n* = 42 (26/no control group) Mean age = 58.4 (6.4)	1 day	1 h	Self-selected	Standalone NW	Lymphoedema	TAV (mL) LAV (mL) LRV (%) VAS (mm)
Casanovas-Álvarez et al. [57]	*n* = 641(30/31) Mean age = 49.2(10.9)/54.7 (12.1)	6–9 weeks	2 t/w 75 min	Borg 6–8	NW combined with resistance training	Adherence Shoulder function and morbidity Shoulder motion Pain Functional capacity Fatigue QoL Physical activity	Quick DASH Arm circumferences Goniometer VAS, 6 MWT, Hand Grip Strength, BFI, EORT QLQ-C30 IPAQ
Vuckovic et al. [64]	*n* = 14/no control group Mean age = 63 (range 58–71)	10 weeks	2 t/w 70–80 min	40–60% HR max	NW combining with concurrent conditioning exercises	Lymphoedema Pain Shoulder motion Strength Aerobic capacity Physical activity	Arm circumferences For point scale Goniometer 30 STS 6 MWT IPAQ
Sartor et al. [65]	*n* = 156 (102 BCS_Meno; 66 BCS_Ind_Meno) Mean age = 58 (6) Age BCS_Meno = 58 (53, 63) Age BCS_Ind_Meno = 46 (43, 48)	12 weeks	3 t/w	Borg 10–14	NW + ISA method	Sedentary behavior Non-exercise physical activity Sleep	Sense Wear Pro2, Pro3 or mini armbands

Intensity prescription was classified according to the method explicitly described by the authors (heart rate-based or Borg RPE). Abbreviations: ACS, memory functioning measured by the Amsterdam Cognitive Series word list learning; BCS_Meno, breast cancer survivor with natural menopause; BCS_Ind_Meno, breast cancer survivor with medically induced menopause; BIPQ, perception of arm and shoulder morbidity measured by the Brief Illness Perception Questionnaire; BFI, fatigue measures by the Brief Fatigue Inventory; BPI, pain measured by the Brief Pain Inventory; CES-D, depressive symptoms measured by the Center for Epidemiologic Studies Depression Scale; DASH, Disability of the Arm, Shoulder and Hand questionnaire; EORT QLQ-C30, quality of life measured by the European Organization for Research and Treatment of Cancer Quality of Life Questionnaire Core 30; HADS, anxiety and depression measured by the Hospital Anxiety and Depression Scale; HVLT-R, cognitive functioning measured by the Hopkins Verbal Learning Test-Revised total recall; ISA, Integrated Systemic Approach method; LAV, lymphedema arm volume; IPAQ, International Physical Activity Questionnaires; LRV, lymphedema reduction volume; MRI, magnetic resonance imaging; NW, Nordic walking; PHQ-9, depressive symptoms measured by the Patient Health Questionnaire-9; PSEQ, pain self-efficacy measured by the Pain Self-Efficacy Questionnaire; QoL, quality of life; SPADI, shoulder pain and disability measured by the Shoulder Pain and Disability Index; STS, Sit to Stand test; TAV, total arm volume; VAS, pain intensity measured by the Visual Analog Scale; VO_2_ peak, peak oxygen uptake.

**Table 4:** Characteristics and results of the studies that analyzed lymphoedema.

Reference	Intervention	Lymphoedema Assessment	Results
Jönsson and Johansson [63]	Number of sessions: 1 Duration: 1 h Type of exercise: 10 min. warm up: slow walking and five exercises for major muscle groups using the poles + 40 min. NW (4 km) + 10 min. cool down: slow walking and stretching exercises. Supervised.	TAV (mL) LAV (mL) LRV (%) VAS (mm)	No significant difference in the TAV in the arm with edema immediately after NOW or 24 h later, compared to before walking. Significant increase in TAV in the healthy arm immediately after NW compared to before walking (*p* = 0.037), but 24 h later this had returned to pre-walking values. Significant decrease in the LAV and LRV immediately after NW compared to before walking. After 24 h, NS compared to measurements from before walking. No significant differences in VAS heaviness and tightness immediately after NW or after 24 h.
Jönsson and Johansen [60]	Weeks: 8 weeks NW + 2 weeks control period Frequency: 3–5 times/week Duration: 30–60 min (excluding warm up and cool down). Intensity: 70–80% of max. HR (200–age)	TAV (mL) LAV (mL) LRV (%) VAS (mm)	Significant reduction in TAV (*p* = 0.001), LAV (*p* = 0.014), LRV (*p* = 0.015). TAV edema T2–T3 - Mean change 95% CI: 51 [22, 80] - TAV healthy T2–T3 - Mean change 95% CI: 16 [−12, 44] - LAV T2–T3 - Mean change 95% CI: 35 [8, 62] - LRV T2–T3 - Mean change 95% C): 1.4 [0.3, 2.5] No significant difference in the reduction in VAS heaviness but significant differences in tightness (*p* = 0.043) VAS tightness T2–T3 - Mean change 95% CI: 6 [0.2, 12]
Malicka et al. [54]	Weeks: 8 Frequency: 60 min/week Type of exercise: - Warm up 10 min = exercise of the upper extremities with poles - NW 40 min - Cool down 10 min = muscle stretching, respiratory and relaxation exercises - Intensity: 85% of max. HR (220–age)	Volume of lymphoedema: 5 levels from the line set by the ulnar styloid process to the radii, continuing every 10 cm up to the shoulder	No significant difference in the reduction in volume of lymphoedema (*p* = 0.39)
Di Blasio et al. [50]	NW group Number of lessons: 10 Frequency: 3 times/week Type of exercise: 15 min. warm up + 35 min. central phase + 10 min. cool down. Warm up and cool down using traditional NW. Association-suggested exercises NW–ISA method Number of lessons: 10 Frequency: 3 times/week Type of exercise: supervised 15 min. warm up + 35 min. central phase + 10 min. cool down Warm up and cool down using ISA method and stretching exercises (cool down did not include a lower limb exercise)	Forearm circumferences → anthropometric tape - relaxed arm - maximal forearm - mid forearm - wrist	NW–ISA method group. Extracellular body water reduced (*p* = 0.001) Extracellular to total water ratio reduced (*p* = 0.001) Circumference of the upper limb (relaxed arm and forearm circumferences) reduced (*p* = 0.001 for all) NW group (no significant difference)
Di Blasio et al. [51]	Weeks: 10 Frequency: 3 times/week Type of exercise: NW NW + ISA method WG W–ISA method Duration: 70 min. 15 min. warm up + 45 min. central phase + 10 min. cool down Intensity: week 1–4→ trained at 10–11 Borg Scale week 5–8 → trained at 12–13 Borg Scale week 9–10 → trained at 13–14 Borg Scale	Forearm circumferences → anthropometric tape - relaxed arm - maximal forearm - mid forearm - wrist	NW + NW–ISA method + W–ISA method. Reduced arm and forearm circumferences homolateral to the surgical intervention (*p* < 0.05) Walking (no significant difference)
Casanovas-Álvarez et al. [57]	Weeks: 6–9 Frequency: 2 days/week Intensity: RPE 6–8 Time + type of exercise: 75 min of NW + muscle strength + health education	Arm circumferences and volume	No significant effect on arm circumference, therefore no effect on lymphoedema indicators compared with control
Vuckovic et al. [64]	Weeks: 10 Frequency: 2 days/week Intensity: 40–60% max HR Time + type of exercise: 70–80 min of NW + strength + stretching	Arm circumferences	NS differences before–after on either side (right arm *p* = 0.326, left arm *p* = 0.087)

Abbreviations: LAV, lymphoedema absolute volume; LRV, lymphoedema relative volume; NW, Nordic walking; TAV, total arm volume; T2, before the exercise intervention; T3, within 3 days of completing the intervention; VAS, visual analog scale.

**Table 5:** GRADE assessment for NW and related exercise interventions.

Outcome	N Studies	Risk of Bias	Heterogeneity and Inconsistency	Indirectness	Imprecision	Publication Bias	Certainty
Range of motion	4	Serious ^a^	Not serious	Serious ^c^	Serious ^d^	Low	Low ^a,c^
Body posture	3	Serious ^a^	Not serious	Serious ^c^	Serious ^d^	Low	Low ^a,c^
Flexibility	1	Serious ^a^	Not serious	Serious ^c^	Serious ^d^	Low	Low ^a,c^
Strength	5	Serious ^a^	Not serious	Serious ^c^	Serious ^d^	Low	Very low ^a,c,d^
Cardiovascular capacity	5	Serious ^a^	Not serious	Serious ^c^	Serious ^d^	Low	Low ^a,c^
Lymphoedema	7	Serious ^a^	Not serious	Serious ^c^	Serious ^d^	Low	Low ^a,c^
Psychological aspects	2	Serious ^a^	Not serious	Serious ^c^	Serious ^d^	Low	Very low ^a,c,d^
Cognitive functioning	2	Serious ^a^	Not serious	Serious ^c^	Serious ^d^	Low	Very low ^a,c,d^
Pain	3	Serious ^a^	Not serious	Serious ^c^	Serious ^d^	Low	Very low ^a,c,d^
Quality of life	5	Serious ^a^	Not serious	Serious ^c^	Serious ^d^	Low	Low ^a,c^

Reason for downgrading: ^a^ study design; ^c^ indirectness; ^d^ imprecision.

## Data Availability

Not applicable.

## Supplementary Materials

The following supporting information can be downloaded at https://www.mdpi.com/article/10.3390/medicina62050932/s1, Supplementary Table S1. Characteristics and results of the studies that analyzed range of motion. Supplementary Table S2. Characteristics and results of the studies that analyzed body posture. Supplementary Table S3. Characteristics and results of the studies that analyzed flexibility and strength. Supplementary Table S4. Characteristics and results of the studies that analyzed cardiovascular fitness. Search strategies and articles retrieved in each database.
