# Supplementary material for: The Physical and Psychological Benefits of Nordic Walking in Patients with Breast Cancer: A Systematic Review
Source: Medicina (Kaunas). 2026 May 11;62(5):932. doi: 10.3390/medicina62050932 (PMC13208209; doi:10.3390/medicina62050932)
Supplement: Supplementary file 1 [file medicina-62-00932-s001.zip › medicina-4256459-supplementary.pdf]

Supplementary Tables.

Supplementary Table S1. Characteristics and results of the studies that analyzed range of motion.

Authors	Sample size (Intervention / Control)	Surgery type (Intervention / Control)	Intervention	Age (Intervention / Control)	ROM Assessment	Results
Sprod et al. (55)	16 (6/6)	Mastectomy (6/5)<br><br>BCT (2/2)<br><br>ALND (3/1)	*Weeks*: 8<br><br>*Frequency*: 2 days/week<br><br>*Intensity*: 40–50% of HR reserve<br><br>*Time + type of exercise*: 20 min. aerobic exercise + 20 min. cardiorespiratory and stretching exercise.	50.33 (2.74) / 59.17 (4.62)	- Baseline goniometer.<br>- Active ROM in flexion, extension, and abduction.	No significant difference was found over time on shoulder press and a significant improvement in muscular endurance as measured by the bench press (Difference pre-post +6.8; p=0.046) and *latissimus dorsi* pull down (Difference pre-post +13; p=0.013).

Fischer et al. (62)	77 (28/no CG)	Mastectomy 20<br><br>Breast conserving surgery + ALND 8	*Weeks*: 10<br><br>*Frequency*: 1 hour/week<br><br>*Type of exercise*:<br>- Sessions 1–6: technique<br>- Session 7–10 NW + endurance + muscle strength	53.8 (10.0)	SPADI<br><br>BIPQ<br><br>Shoulder ROM assessed using a goniometer.	SPADI<br>- Pain decrease<br>T1−T2 no significant differences<br>T1−T3 ($p < 0.01$)<br>- Disability decrease<br>T1−T2 no significant differences<br>T1−T3 ($p < 0.01$)<br><br>BIPQ<br>Consequences and symptoms decrease<br>T1−T2 ($p < 0.05$)<br>T1−T3 ($p < 0.05$)<br><br>ROM<br>Active motion increase ($p < 0.01$)<br>Passive motion increase ($p < 0.05$)

Casanovas-Álvarez et al (57)	64 (30/31)	Tumorectomy ALND (7/5)<br><br>Tumorectomy SLNB (6/7)<br><br>Radical mastectomy ALND (11/6)<br><br>Radical mastectomy SLNB (2/8)<br><br>Surgery without node extirpation (4/5)	*Weeks*: 6-9<br><br>*Frequency*: 2 days/week<br><br>*Intensity*: RPE 6-8<br><br>*Time + type of exercise*: 75 min of NW + muscle strength + health education	49.2(10.9)/54.7 (12.1)	Shoulder Rom ◎ manual goniometer<br><br>Quick DASH	ROM decreased<br>- NS differences between groups<br>- Control group lost >10% degrees of shoulder flexion compared to prehab group (20 vs 11, p=0.048) 1 month after surgery<br><br>DASH<br>-Significant effect of the surgery worsening at T2 (z=5.952, p<0.001) and T3 (z=3.020, p=0.002)<br>- Significant interaction between TxGroup at T1 (p=0.058)<br>- Positive impact of the prehab group before surgery (p=0.025)

Vuckovic et al (64)	14	Surgical treatment 17/14 (bilateral involvement and 2 required reinterventions on the affected upper limb)	*Weeks*: 10<br><br>*Frequency*: 2 days/week<br><br>*Intensity*: 40-60% max HR<br><br>*Time + type of exercise*: 70-80 min of NW+ strength+ stretching	63(58-71)	ROM: manual goniometer	Improved in all areas except for left anteflexion (p=0.055) and left retroflexion (p==0.051)

Abbreviations: ALND, axillary lymph node dissection; BCT, breast conserving therapy; BIPQ, perception of arm and shoulder morbidity measured by the *Brief Illness Perception Questionnaire*; DASH, Disabilities of the Arm, Shoulder and Hand questionnaire; HR, heart rate; NW, Nordic walking; ROM, range of motion; RPE, Rating of Perceived Exertion; SLNB, Sentinel Lymph node Biopsy; SPADI, shoulder morbidity evaluated with the *Shoulder Pain and Disability Index*; T1, before starting the NW program; T2, after the 10th training session; T3, 6 months after the end of the course.

Supplementary Table S2. Characteristics and results of the studies that analyzed body posture.

Authors	Simple size (Intervention / Control)	Surgery type (Intervention / Control)	Intervention	Age (Intervention / Control)	Body Posture Assessment	Results
Hanuszkiewicz et al. (58)	60<br>(NW = 20/GE = 20/WE = 20)	Mastectomy	*Weeks*: 8<br><br>*Frequency*: 2 sessions/week<br>*Duration*: 45 min/session<br><br>*Intensity*: 70–75% max. HR (220–age)<br><br>*Type of exercise*: Fitness walking with poles<br>from 2,400 m weeks 1–2 to 3,600 weeks 7–8.	NW = 57.3 (8.05)<br>GE = 59.4 (7.47)<br>WE = 63.0 (7.58)	Trunk flexors and extensors using an isokinetic test.	Intragroup improvement in muscle work and power.<br><br>Intergroup improvement compared with the general exercise group.

Hanuszkiewicz et al (59)	60<br>(NW = 20/GE = 20/WE = 20)	Mastectomy	*Weeks*: 8<br><br>*Frequency*: 2 sessions/week<br><br>*Duration*: 45 min/session<br><br>*Intensity*: 70−75% max. HR (220−age)<br><br>*Type of exercise*: Fitness walking with poles<br>from 2,400 m weeks 1−2 to 3,600 weeks 7−8.	NW = 57.3 (8.05)<br>GE = 59.4 (7.47)<br>WE = 63.0 (7.58)	Body posture using a photogrammetric body posture test.	Significant intragroup changes in angular parameters.<br><br>Intergroup thoracolumbar inclination decreases; trunk inclination increase.

Hanuszkiewicz et al (56)	58 (19/20)	82% mastectomy<br>18% BCT	*Weeks*: 8<br><br>*Frequency*: 2 sessions/week<br><br>*Type of exercise*:<br>- Supervised<br>- 45 min (5 min. warm up, 35 min. main part, 5 min. cool down).<br>- Sessions 1–4 → 6 × 400 = 2,400 m + 5 × 30 s rest period<br>- Sessions 5–8 → 5 × 560 = 2,800 m + 4 × 30 s rest period<br>- Sessions 9–12 → 4 × 800 = 3,200 m + 3 × 30 s rest period<br>- Sessions 13–16 → 3 × 1,200 = 3,600 + 2 × 30 s rest period	58.8 (7.30)<br>NW < 60 = 53.09 (4.57)<br>NW > 60 = 65.25 (2.82)<br><br>GG < 60 = 52.7 (4.46)<br>GG > 60 = 64.82 (4.28)	Trunk muscle endurance testing → Biodex multi joint 3 isokinetic dynamometer.<br><br>Anterior and posterior curvatures of the spine using a CQ Elektronik System.	NW in middle-aged patients significantly reduced the size of thoracic kyphosis.<br><br>NW in middle-aged patients significantly increased trunk flexion and extensor muscle endurance.<br><br>NW in older women significantly increased flexor muscle endurance.

Abbreviations: BCT, breast conserving therapy; GE, general exercise; GG: general gymnastic exercise; HR, heart rate; NW, Nordic walking; WE, water exercise

Supplementary Table S3. Characteristics and results of the studies that analyzed flexibility and strength.

Authors	Simple size (Intervention / Control)	Surgery type (Intervention / Control)	Intervention	Age (Intervention / Control)	Body Posture Assessment	Results
Morano et al. (61)	160 (NW = 49/ME = 70)	Quadrantectomy (16/21)<br>Resection (20/29)<br>Mastectomy (13/20)<br>Lymphectomy (17/14)	Weeks: 12<br><br>Frequency: 3 times/week<br><br>Type of exercise:<br>10 NW technique lessons + 26 lessons of complete technique + ISA method.<br><br>15 min. warm up (ISA method) 45 min. central phase 10 min. cool down (ISA method + stretching).	52.85 (7.26)<br>[49.24 (5.55) /54.49 (7.43)]	FLEXIBILITY<br>BST→ shoulder joint and shoulder arc flexibility.<br><br>SRT→ lower back and hamstring musculature flexibility.<br><br>STRENGTH<br>SLBBT→ lumbar multifidus and erector spinae muscle.<br><br>HG test→ maximum isometric strength of the hand and forearm muscle	Significant changes in flexibility (right and left back scratch and sit and reach test [p<0.001])<br><br>Significant changes in strength (right and left HG, total HG, right and left single-leg back bridge [p<0.001])<br><br>NS when data were analyzed by sub-sample. Age seemed to negatively affect left back scratch test (p=0.02) and sit and reach (p=0.002). Radiation therapy seemed to affect the right back scratch test (p = 0.04) while chemotherapy seemed to affect the single-leg back bridge test (right p = 0.001, left p = 0.004).

Sprod et al. (55)	16(6/6)	Mastectomy (6/5)<br>BCT (2/2)<br>ALND (3/1)	Weeks: 8<br><br>Frequency: 2 days/week<br><br>Intensity: 40−50% of HR reserve.<br><br>Time + type of exercise: 20 min. aerobic exercise + 20 min. cardiorespiratory and stretching exercise.	50.33 (2.74) /59.17 (4.62)	30 repetitions/minute.<br><br>Participants were asked to perform as many repetitions as possible before volitional muscular fatigue.<br><br>Bench press.<br>Shoulder press.<br>Latissimus dorsi pull down.	Bench press.<br>Difference from pre to post exercise intervention 6.83 (p = 0.046).<br><br>Shoulder press.<br>Difference from pre to post exercise intervention 1.17 (no significant difference).<br><br>Latissimus pull down.<br>Difference from pre to post exercise intervention 13<br>p = 0.013.

Malicka et al. (54)	38<br>(23/15)	Radical mastectomy (82.6%/73.3%)<br>BCT (17.4%/26.7%)	Weeks: 8<br><br>Frequency: 60 min/week<br><br>Type of exercise:<br>- Warm up 10 min. = exercise of the upper extremities with poles.<br>- NW 40 min.<br>- Cool down 10 min. = muscle stretching, respiratory and relaxation exercises.<br>Intensity: 85% of max. HR (220−age).	62.8 (6.1)<br>[63.6 (6.8)/63.8 (9.2)]	Muscle strength→ dynamometer (Biodex multi joint 3 isokinetic)<br><br>3 tests × 5 rep<br>- 36.67 cm/s<br>- 24.44 cm/s<br>12.22 cm/s	Significant changes in the strength of the pushing motion of both the right and left was found in women after the surgical procedure on the right side.<br><br>In women after the surgical procedure on the left side, only significant changes were found on the left extremity.

Casanovas-Álvarez et al (57)	64 (30/31)	Tumorectomy ALND (7/5)<br><br>Tumorectomy SLNB (6/7)<br><br>Radical mastectomy ALND (11/6)<br><br>Radical mastectomy SLNB (2/8)<br><br>Surgery without node extirpation (4/5)	*Weeks*: 6-9<br><br>*Frequency*: 2 days/week<br><br>*Intensity*: RPE 6-8<br><br>*Time + type of exercise*: 75 min of NW + muscle strength + health education	49.2(10.9)/54.7 (12.1)	HG with Jamar dynamometer	Significant effects at T3 (z=-2.050, p=0.040) ◎ decline over time<br>NS changes between groups or TxGroup interaction<br>Control group significant decline from baseline to 1month after surgery (T2) (p=0.036)

Vuckovic et al (64)	14	Surgical treatment 17/14 (bilateral involvement and 2 required reinterventions on the affected upper limb)	*Weeks*: 10<br><br>*Frequency*: 2 days/week<br><br>*Intensity*: 40-60% max HR<br><br>*Time + type of exercise*: 70-80 min of NW+ strength+ stretching	63(58-71)	HG<br>30STS	HG<br>Improvement in left hand after intervention (p=0.007)<br><br>30STS improved after intervention (p=0.006)

Abbreviations: ALND, axillary lymph node dissection; BST, Back Scratch Test; BCT, breast conserving therapy; HG, Hand Grip; HR, heart rate; ME, myofascial exercise; NW, Nordic walking; SLBBT, Single leg back bridge test; SRT, Sit and reach test; 30 STS, 30 sec Sit to Stand Test

Supplementary Table S4. Characteristics and results of the studies that analyzed cardiovascular fitness.

Authors	Simple size (Intervention / Control)	Surgery type (Intervention / Control)	Intervention	Age (Intervention / Control)	Body Posture Assessment	Results
Jönsson and Johansen (60)	35<br><br>(23/no CG)	ALND with or without radiotherapy	*Weeks*: 8 weeks NW + 2 weeks control period.<br><br>*Frequency*: 3–5 times/week<br><br>*Duration*: 30–60 minutes (excluding warm up and cool down).<br><br>*Intensity*: 70–80% of max. HR (200–age).	60.4 (8.3)/no CG	Cardiovascular fitness→ bicycle ergometer test.	Cardiovascular fitness→ significant decrease T2–T3 (mean change 5 (95% CI: [1.4, 6.5], $p = 0.004$).

Koevoets et al. (52)	3,258<br><br>(91/90)	Surgery<br><br>(91/89)	*Months*: 6<br><br>*Frequency*: 4h/week<br><br>*Duration and type of exercise*: 2 h/week supervised aerobic and strength training + 2 h/week NW.	52<br><br>52.1 (8.6)/52.5 (8.7)	VO_2 peak→ cycle cardiopulmonary exercise test.	Significant increase from baseline to follow-up in physical fitness in the IG vs. CG.<br><br>B VO_2 peak<br><br>1.39 mL/min/kg, (95% CI: [0.59, 2.19], ES = 0.26).

Koevoets et al. (53)	3,258<br><br>(70/72)	Surgery<br><br>(70/72)	*Months*: 6<br><br>*Frequency*: 2 times/week<br><br>*Duration and type of exercise*: 1 h supervised aerobic and strength training + 1 h NW.	52.5 (9.9)/53.2 (8.6)	Physical fitness→ maximal cycle CPET including continuous breathing gas analysis and electrocardiogram monitoring.<br><br>Over the final 30 s of exercise, the average for relative maximum oxygen uptake (VO2 peak) divided by baseline body weight (in kg) was calculated.	Physical fitness improved after the intervention in comparison to the CG (B = 1.40 mL/min/kg, 95% CI: [0.55, 2.26], ES = 0.26).

Casanovas-Álvarez et al (57)	64 (30/31)	Tumorectomy ALND (7/5)<br><br>Tumorectomy SLNB (6/7)<br><br>Radical mastectomy ALND (11/6)<br><br>Radical mastectomy SLNB (2/8)<br><br>Surgery without node extirpation (4/5)	*Weeks*: 6-9<br><br>*Frequency*: 2 days/week<br><br>*Intensity*: RPE 6-8<br><br>*Time + type of exercise*: 75 min of NW + muscle strength + health education	49.2(10.9)/54.7 (12.1)	6MWT	Prehab group improved along the time before surgery<br>- T0: 592.13 m; p=0.001<br>- T1: 638. 87 m; p<0.001<br>- T2: 607.97 m; p<0.001<br>- T3: 613.53 m; p<0.001

Vuckovic et al (64)	14	Surgical treatment 17/14 (bilateral involvement and 2 required reinterventions on the affected upper limb)	*Weeks*: 10<br><br>*Frequency*: 2 days/week<br><br>*Intensity*: 40-60% max HR<br><br>*Time + type of exercise*: 70-80 min of NW+ strength+ stretching	63(58-71)	6MWT	6MWT improved after intervention (p=0.044)

Abbreviations: ALND, axillary lymph node dissection; CG, control group; CPET, cardiopulmonary exercise test; ES, effect size*; HR, heart rate; IG, intervention group; NW, Nordic walking; T1, before control period; T2, before the exercise intervention; T3, within 3 days of completing the intervention; VO2 peak, relative maximum oxygen uptake. *ES < 0.2 no differences; ES = 0.2–0.5 small differences; ES = 0.5–0.8 medium differences; ES ≥ 0.8 large differences; 6MWT, 6 Minutes Walking Test.

Search strategies and articles retrieved in each database

Sport Discuss: 0 Results

CINHAL: Results

1) “Breast Cancer” AND “Walking Poles” (1 Results)
2) “Breast Cancer” AND “Pole Walking” (3 Results)
3) “breast cancer” AND (“walking poles” OR “pole walking” OR “Nordic walking”) 17 Results

Cochrane:

1) Breast Cancer AND Nordic Walking OR “Pole Walking” (169 Results)

EMBASE

1) ('Breast Cancer'/exp OR 'Breast Cancer') AND ('Nordic Walking'/exp OR 'Nordic Walking') (48 Results)
2) ('Breast Cancer'/exp OR 'Breast Cancer') AND ('Pole Walking'/exp OR 'Pole Walking') (3 Results)

MEDLINE Ovid

1) Breast Cancer AND Nordic Walking (9 Results)
2) Breast Cancer AND Pole Walking (2 Results)
3) “breast cancer” AND (“walking poles” OR “pole walking” OR “Nordic walking”) 12 Results

PUBMED

1) “breast cancer” AND (“walking poles” OR “pole walking” OR “Nordic walking”) 18 Results

Total= 282
